# The Lipid Paradox is present in ST-elevation but not in non-ST-elevation myocardial infarction patients: Insights from the Singapore Myocardial Infarction Registry

**DOI:** 10.1038/s41598-020-63825-8

**Published:** 2020-04-22

**Authors:** Ching-Hui Sia, Huili Zheng, Andrew Fu-Wah Ho, Heerajnarain Bulluck, Jun Chong, David Foo, Ling-Li Foo, Patrick Zhan Yun Lim, Boon Wah Liew, Huay-Cheem Tan, Tiong-Cheng Yeo, Terrance Siang Jin Chua, Mark Yan-Yee Chan, Derek J. Hausenloy

**Affiliations:** 1 Department of Cardiology, National University Heart Centre, Singapore, Singapore; 2 Yong Loo Lin School of Medicine, National University of Singapore, Singapore, Singapore; 3 Health Promotion Board, National Registry of Diseases Office, Singapore, Singapore; 4 SingHealth Duke-NUS Emergency Medicine Academic Clinical Programme, Singapore, Singapore; 5 Cardiovascular & Metabolic Disorders Program, Duke-NUS Medical School, Singapore, Singapore; 6 National Heart Research Institute Singapore, National Heart Centre, Singapore, Singapore; 7 Norfolk and Norwich University Hospital, Norwich, United Kingdom; 8 Tan Tock Seng Hospital, Singapore, Singapore; 9 KhooTeck Puat Hospital, Singapore, Singapore; 10 Changi General Hospital, Singapore, Singapore; 11 Department of Cardiology, National Heart Centre, Singapore, Singapore; 12 The Hatter Cardiovascular Institute, University College London, London, United Kingdom; 13 Cardiovascular Research Center, College of Medical and Health Sciences, Asia University, Taichung City, Taiwan

**Keywords:** Cardiology, Acute coronary syndromes, Dyslipidaemias

## Abstract

Lowering low-density lipoprotein (LDL-C) and triglyceride (TG) levels form the cornerstone approach of cardiovascular risk reduction, and a higher high-density lipoprotein (HDL-C) is thought to be protective. However, in acute myocardial infarction (AMI) patients, higher admission LDL-C and TG levels have been shown to be associated with better clinical outcomes - termed the ‘lipid paradox’. We studied the relationship between lipid profile obtained within 72 hours of presentation, and all-cause mortality (during hospitalization, at 30-days and 12-months), and rehospitalization for heart failure and non-fatal AMI at 12-months in ST-segment elevation myocardial infarction (STEMI) and non-ST-segment elevation myocardial infarction (NSTEMI) patients treated by percutaneous coronary intervention (PCI). We included 11543 STEMI and 8470 NSTEMI patients who underwent PCI in the Singapore Myocardial Infarction Registry between 2008–2015. NSTEMI patients were older (60.3 years vs 57.7 years, p < 0.001) and more likely to be female (22.4% vs 15.0%, p < 0.001). In NSTEMI, a lower LDL-C was paradoxically associated with worse outcomes for death during hospitalization, within 30-days and within 12-months (all p < 0.001), but adjustment eliminated this paradox. In contrast, the paradox for LDL-C persisted for all primary outcomes after adjustment in STEMI. For NSTEMI patients, a lower HDL-C was associated with a higher risk of death during hospitalization but in STEMI patients a lower HDL-C was paradoxically associated with a lower risk of death during hospitalization. For this endpoint, the interaction term for HDL-C and type of MI was significant even after adjustment. An elevated TG level was not protective after adjustment. These observations may be due to differing characteristics and underlying pathophysiological mechanisms in NSTEMI and STEMI.

## Introduction

Acute myocardial infarction (AMI) and the heart failure which often follows are among the leading causes of death and disability worldwide^1^. Elevated levels of low-density lipoprotein (LDL-C) and triglycerides (TG) are well-established risk factors for developing AMI^2–4^. Circulating LDL-C enters the endothelium of arterial walls resulting in inflammation and formation of atherosclerotic plaques, which on rupturing,result in AMI^3^. Similarly, elevated TG levels are known to cause premature atherosclerosis^5^. Pharmacological lowering of LDL-C and TG levels can prevent atherosclerotic disease and subsequent AMI^6^. Although, a low level of high-density lipoprotein cholesterol (HDL-C) is a known risk factor for the development of AMI^7^, pharmacological treatments aimed at elevating HDL-C levels have not been shown to improve clinical outcomes^8^.

Despite these well-established associations, some studies have described the existence of a ‘lipid paradox’ in AMI patients. These patients paradoxically have better outcomes despite having higher LDL-C and TG levels at time of admission. Previous studies have studied the lipid paradox in AMI patients, but did not specifically examine the phenomenon of the lipid paradox between non-ST elevation myocardial infarction (NSTEMI) and STEMI^9–13^. While there are some similarities between the pathophysiology underlying STEMI and NSTEMI populations^14^, STEMI populations have been found to have an increased pro-inflammatory state and a different serological profile compared to NSTEMI patients^15–17^.

We hypothesize that there are differences in the lipid paradox between STEMI and NSTEMI patients and this may be attributed to the differences in underlying pathophysiology between the 2 entities. As such, we conducted this study to clarify the relationship of the lipid paradox and clinical outcomes amongst STEMI and NSTEMI patients who have had percutaneous coronary intervention (PCI) using a nationwide AMI registry.

## Methods

We conducted a retrospective observational analysis of patients with AMI from our national registry, The Singapore Myocardial Infarction Registry (SMIR). This registry is managed by the National Registry of Diseases Office and collects epidemiological and clinical data on all AMI cases diagnosed in all public and private sector hospitals and a small number of out-of-hospital AMI deaths certified by medical practitioners in Singapore^18–20^. Notification of AMI to the registry has been mandated by the National Registry of Diseases Act enacted in 2012. Public sector cases comprised 98% of the registered cases. Registry data were received from various sources and were processed to obtain unique cases. The sources of data included patient medical claim listings, hospital in-patient discharge summaries, cardiac biomarker listings from hospital laboratories and the national death registry. The International Classification of Diseases, Ninth Revision, Clinical Modification (ICD-9-CM) code 410 was used to identify AMI cases diagnosed prior to 2012 while ICD-10 (Australian Modification) codes I21 and I22 were used for AMI cases diagnosed in 2012. The differentiation between STEMI and NSTEMI was based on presenting symptoms, cardiac biomarkers and ECG assessment, and aligned with clinician’s diagnosis documented in the physical case notes and electronic medical records. STEMI was defined as follows: typical chest pain of 20 minutes and significant ST-segment elevation (0.1 or 0.2 mV on 2 adjacent limb or precordial leads, respectively, or new left bundle-branch block) and confirmed subsequently by a rise in biomarkers. All ECGs were interpreted, and all diagnoses were adjudicated centrally at the National Registry of Diseases Office. The multinational monitoring of trends and determinants in cardiovascular disease (MONICA) criteriawere used for defining episodes. Detailed patient data were extracted from clinical medicalrecords including Emergency department notes, clinical charts, and discharge summaries, by dedicated registry coordinators from the SMIR. Yearly audits on data collected were done to ensure data accuracy and inter-rater reliability. Logic checks were done and illogical or outlier data were highlighted for review.

This study was based onAMI cases reported to the SMIR from 1^st^ January 2008 onwards who had PCI. Patients without PCI were excluded as we wanted to study those patients with a Type 1 myocardial infarction^21^.

The exposure of interest was lipid profile obtained within 72 hours of the AMI (LDL-C, Total cholesterol [TC]; high-density lipoprotein [HDL-C]; TG). The lipid profile was analyzed in both numeric and categorical form. Lipids were categorized based on local guidelines^22^. LDL-C levels were divided into optimal (1.8–2.5 mmol/L), desirable (2.6–3.3 mmol/L), borderline high (3.4–4.0 mmol/L), high (4.1–4.8 mmol/L) and very high (≥4.9 mmol/L) levels; TC levels into desirable (<5.2 mmol/L), borderline high (5.2–6.1 mmol/L) and high (≥6.2 mmol/L) levels; HDL-C levels into low (<1.0 mmol/L), desirable (1.0–1.5 mmol/L) and optimal (≥1.6 mmol/L) levels; TG levels into optimal (<1.7 mmol/L), desirable (1.7–2.2 mmol/L), high (2.3–4.4 mmol/L) and very high (≥4.5 mmol/L) levels. A stricter cut-off of 1.8 mmol/L for LDL-C was used in line with international guidelines as there was the most evidence for risk modification by lowering LDL-C compared to the other cholesterol fractions^23,24^. Primary outcomes of interest were all-cause mortality during hospitalization, within 30-day and within 12 months. As the mortality data, obtained from the Death Registry of Ministry of Home Affairs, was available until 2016 at the point of analysis, findings on primary outcomes were based on AMI cases with onset in 2008 to 2015. Secondary outcomes were rehospitalization within 12 months for heart failure (HF)andAMI for patients that were discharged alive. As the rehospitalization data, obtained from the final discharge diagnosis registered with the national financial claims database of Ministry of Health, was available until 2014 at the point of analysis, findings on secondary outcomes were based on AMI cases with onset in 2008 to 2013. No patient was lost to follow-up as it is mandatory for all deaths to be registered within 24 hours of occurrence^25^ and the national financial claims database covers all public and private healthcare institutions.

We analyzed the AMI cases as a whole and had comparison groups comprising STEMI and NSTEMI patients. Numeric variables were expressed as median and interquartile range, while categorical variables were expressed as frequency and percentages. Comparison between STEMI and NSTEMI patients was done using Wilcoxon rank sum test for numeric variables and Chi-square test for categorical variables. Cox regression was used to estimate the risk of death during hospitalization, death within 30 days of AMI onset and death within 1 year of AMI onset. Coxregressionadjusted for the competing risk from death was used to estimate the risk of hospitalization for HF and AMI within 1 year from AMI discharge. Multivariable models were adjusted for whether patient was on oral medication for hyperlipidemia, age, sex, race, body mass index, history of diabetes, history of hypertension, smoking status, history of AMI/coronary artery bypass grafting (CABG)/PCI, Killip class on admission, presence of cardiopulmonary resuscitation (CPR) in ambulance, random blood glucose levels within 72 hours of onset of AMI, admission creatinine, admission haemoglobin, presence of elevated first troponin within 72 hours from AMI onset, left ventricular ejection fraction (LVEF) of <50% during hospitalization, presence of anterior myocardial infarction (for STEMI only), and symptom-to-balloon time (for STEMI only). These variables were selected to be included in the multivariable models as they were statistically different across the categories of lipids and they were clinically associated with the outcomes of interest. We performed further analyses to study the interaction between the type of MI (STEMI/NSTEMI) and lipids in relation to the primary and secondary outcomes. Supplementary Fig. 1 shows the patient selection criteria. A sensitivity analysis for missing data was also performed. This was done using multiple imputation with 20 imputed datasets and no auxiliary variables based on the Markov Chain Monte Carlo procedure assuming all variables in the model having a joint multivariate normal distribution. Sensitivity analysis showed that the results were in the similar direction albeit with differing magnitudes and statistical significance. As such, we opted to maintain the data in its original form and missing data were dropped from analyses through case deletion without any imputation.

The institutional review board granted an exemption for conducting this study without need for informed consent (SingHealth Centralised Institutional Review Board Reference No: 2016/2480) as this study involved analysis of a dataset without identifiers. The research was conducted in accordance with the Declaration of Helsinki. The statistician had access to anonymizedindividual data points while the other co-authors had access to analyzed, aggregated data. Statistical analysis was performed using Stata SE Version 13 (StataCorp. 2013. Stata Statistical Software: Release 13. College Station, TX: StataCorp LP). All reported p-values were 2-sided and p-values < 0.05 were considered to be statistically significant.

## Results

The final patient population comprised 20013 patients. There were 11543 STEMI patients and 8470 NSTEMI patients available for analysis. Patient characteristics are shown in Table 1. STEMI patients were about 2 years younger than NSTEMI patients and more likely to be male. Fewer STEMI patients had a prior history of diabetes mellitus, hypertension, history of AMI/CABG/PCI but they were more likely to be smokers. In terms of cholesterol levels, STEMI patients had a higher LDL-C (3.4 mmol/l vs 3.2 mmol/l, p < 0.001), lower TG levels (1.4 mmol/l vs 1.6 mmol/l, p < 0.001) but there was no difference in HDL-C levels. There were a higher proportion of STEMI patients than NSTEMI patients with higher TC. STEMI patients were less likely to be on oral medications for hyperlipidemia prior to presentation (62.6% vs 75.1%, p < 0.001). STEMI patients were also more likely to have a depressed ejection fraction (61.2% vs 39.6%, p < 0.001).

**Table 1:** Characteristics of ST elevation (STEMI) and non-ST elevation myocardial infarction (NSTEMI) patients in the study.

	STEMI (n = 11543)	NSTEMI (n = 8470)	p
**Age in years, median (IQR)**	57.7 (50.7–66.0)	60.3 (52.5–69.4)	<0.001
**Sex, n (%)**			
Male	9810 (85.0)	6570 (77.6)	<0.001
Female	1733 (15.0)	1900 (22.4)	
**Race, n (%)**			
Chinese	7125 (61.7)	5253 (62.0)	0.004
Malay	2343 (20.3)	1575 (18.6)	
Indian	1874 (16.2)	1499 (17.7)	
Others	201 (1.7)	143 (1.7)	
**History of diabetes, n (%)**			
Yes	3252 (28.2)	3312 (39.1)	<0.001
No	8285 (71.8)	5156 (60.9)	
**History of hypertension, n (%)**			
Yes	6014 (52.1)	5730 (67.7)	<0.001
No	5523 (47.9)	2739 (32.3)	
**Smoking, n (%)**			
Never	4203 (36.8)	3689 (43.7)	<0.001
Former	1571 (13.8)	1657 (19.6)	
Current	5640 (49.4)	3093 (36.7)	
**History of AMI/CABG/PCI, n (%)**			
Yes	1744 (15.1)	2733 (32.3)	<0.001
No	9797 (84.9)	5737 (67.7)	
**BMI in kg/m**^**2**^**, median (IQR)**	24.6 (22.3–27.3)	24.9 (22.6–27.9)	<0.001
**Killip class on admission, n (%)**			
I	9549 (82.7)	7061 (83.4)	<0.001
II	543 (4.7)	805 (9.5)	
III	436 (3.8)	495 (5.8)	
IV	1014 (8.8)	107 (1.3)	
**CPR in ambulance/ED, n (%)**			
Yes	474 (4.1)	50 (0.6)	<0.001
No	11069 (95.9)	8420 (99.4)	
**Anterior infarct on admission, n (%)**			
Yes	5756 (49.9)	Not applicable	
No	5787 (50.1)		
**LDL-C in mmol/l within 72 h from MI onset**			
Numeric value, median (IQR)	3.4 (2.6–4.1)	3.2 (2.4–4.0)	<0.001
Ordinal categories, n (%)			
<1.8	707 (6.8)	652 (9.2)	<0.001
1.8–2.5	1881 (18.1)	1598 (22.6)	
2.6–3.3	2719 (26.1)	1771 (25.1)	
3.4–4.0	2491 (23.9)	1418 (20.1)	
4.1–4.8	1623 (15.6)	970 (13.7)	
≥4.9	1002 (9.6)	653 (9.2)	
**TC in mmol/lwithin 72 h from MI onset**			
Numeric value, median (IQR)	5.1 (4.3–6.0)	5.0 (4.1–5.9)	<0.001
Ordinal categories, n (%)			
<5.2	5768 (54.2)	4153 (57.3)	<0.001
5.2–6.1	2854 (26.8)	1697 (23.4)	
≥6.2	2026 (19.0)	1397 (19.3)	
**HDL-C in mmol/l within 72 h from MI onset**			
Numeric value, median (IQR)	1.0 (0.9–1.2)	1.0 (0.9–1.2)	0.261
Ordinal categories, n (%)			
≥1.6	510 (4.8)	363 (5.0)	0.056
1.0–1.5	5275 (49.5)	3455 (47.7)	
<1.0	4874 (45.7)	3430 (47.3)	
**TG in mmol/l within 72 h from MI onset**			
Numeric value, median (IQR)	1.4 (1.0–2.0)	1.6 (1.1–2.3)	<0.001
Ordinal categories, n (%)			
<1.7	6917 (65.1)	4020 (55.8)	<0.001
1.7–2.2	1874 (17.6)	1496 (20.8)	
2.3–4.4	1543 (14.5)	1442 (20.0)	
≥4.5	289 (2.7)	247 (3.4)	
**Oral medication for hyperlipidemia, n (%)**			
Yes	3315 (62.6)	4093 (75.1)	<0.001
No	1983 (37.4)	1359 (24.9)	
Not applicable	6235	3017	
**Random glucose in mmol/L within 72 h from MI onset, median (IQR)**	9.1 (7.2–13.2)	8.2 (6.3–12.5)	<0.001
**Serum creatinine in 10µmol on admission, median (IQR)**	9.0 (7.7–10.9)	8.6 (7.3–10.8)	<0.001
**Haemoglobin in g/dL on admission, median (IQR)**	14.6 (13.4–15.7)	14.0 (12.6–15.2)	<0.001
**Elevated first troponin within 72 h from MI onset, n (%)**			
Yes	5585 (49.3)	4655 (55.5)	<0.001
No	5745 (50.7)	3730 (44.4)	
**Symptom-to-balloon time in minutes, median (IQR)**	182 (119–307)	Not applicable	
**Days from MI onset to PCI, median (IQR)**	0 (0–0)	2 (1–4)	<0.001
**LVEF** < **50% during hospitalization, n (%)**			
Yes	6610 (61.2)	2742 (39.6)	<0.001
No	4195 (38.8)	4176 (60.4)	
**Days from MI onset to discharge, median (IQR)**	4 (3–5)	4 (3–6)	<0.001

Abbreviations: AMI, acute myocardial infarction; BMI, body mass index; CPR, cardiopulmonary resuscitation; CABG, coronary artery bypass grafting; ED, emergency department; HDL-C, high density lipoprotein cholesterol; IQR, interquartile range; LDL-C, low density lipoprotein cholesterol; LVEF, left ventricular ejection fraction; PCI, percutaneous coronary intervention; TC, total cholesterol; TG, triglycerides.

### AMI Population

Firstly, we analyzed the entire AMI population. Higher LDL-C levels were associated with a lower risk of death during hospitalization, at 30 days and at 1 year on both unadjusted and adjusted analysis (Table 2) but not for rehospitalization for heart failure or myocardial infarction within 1 year from discharge. While there was a similar association for TG levels for the risk of death, this did not persist after adjustment (Supplementary Table 1). There was an association between higher TC levels with worse primary outcomes on unadjusted and adjusted analysis for the primary outcomes but not for the secondary outcomes of interest (Supplementary Table 2). After adjustment, there was actually a higher risk of death at 30-days and at 1-year with HDL-C levels of <1.0 mmol/L which suggest that there was no paradox present for HDL-C levels for the entire AMI population (Supplementary Table 3).

**Table 2:** Unadjusted and adjusted analysis examining the correlations between low density lipoprotein cholesterol levels and the primary and secondary outcomes in ST elevation myocardial infarction (STEMI) and Non-ST elevation myocardial infarction (NSTEMI) patients who underwent percutaneous coronary intervention.

	All STEMI + NSTEMI patients						All STEMI + NSTEMI patients discharged alive			
	Death during hospitalization		Death within 30 days from MI onset		Death within 1 year from MI onset		Rehospitalization for HF within 1 year from MI discharge		Rehospitalization for MI within 1 year from MI discharge	
	HR (95% CI)	p	HR (95% CI)	p	HR (95% CI)	p	HR (95% CI)	p	HR (95% CI)	p
**Unadjusted**										
LDL-C in mmol/l	**0.77 (0.74–0.80)**	<0.001	**0.65 (0.62–0.68)**	<0.001	**0.68 (0.66–0.70)**	<0.001	0.97 (0.90–1.04)	0.411	0.95 (0.89–1.03)	0.220
**Ordinal categories**										
<1.8	1.00 (ref)		1.00 (ref)		1.00 (ref)		1.00 (ref)		1.00 (ref)	
1.8–2.5	**0.68 (0.60–0.78)**	<0.001	**0.52 (0.46–0.58)**	<0.001	**0.58 (0.53–0.62)**	<0.001	0.90 (0.68–1.19)	0.451	1.05 (0.76–1.44)	0.783
2.6–3.3	**0.56 (0.49–0.65)**	<0.001	**0.37 (0.32–0.42)**	<0.001	**0.40 (0.36–0.44)**	<0.001	0.95 (0.72–1.25)	0.719	1.04 (0.76–1.42)	0.821
3.4–4.0	**0.45 (0.38–0.53)**	<0.001	**0.26 (0.22–0.30)**	<0.001	**0.29 (0.26–0.32)**	<0.001	0.76 (0.56–1.03)	0.078	0.86 (0.61–1.22)	0.402
4.1–4.8	**0.47 (0.38–0.58)**	<0.001	**0.25 (0.21–0.31)**	<0.001	**0.26 (0.23–0.30)**	<0.001	0.77 (0.55–1.08)	0.130	0.89 (0.61–1.30)	0.553
≥4.9	**0.37 (0.29–0.48)**	<0.001	**0.22 (0.15–0.33)**	<0.001	**0.32 (0.28–0.37)**	<0.001	0.95 (0.66–1.36)	0.775	0.88 (0.57–1.34)	0.543
Adjusted*										
LDL in mmol/l	**0.81 (0.76–0.87)**	<0.001	**0.79 (0.74–0.85)**	<0.001	**0.88 (0.84–0.91)**	<0.001	1.01 (0.93–1.10)	0.795	0.98 (0.88–1.08)	0.640
**Ordinal categories**										
<1.8	1.00 (ref)		1.00 (ref)		1.00 (ref)		1.00 (ref)		1.00 (ref)	
1.8–2.5	**0.72 (0.60–0.86)**	<0.001	**0.62 (0.52–0.73)**	<0.001	**0.77 (0.68–0.86)**	<0.001	0.83 (0.59–1.16)	0.281	1.14 (0.76–1.70)	0.519
2.6–3.3	**0.62 (0.50–0.76)**	<0.001	**0.54 (0.44–0.66)**	<0.001	**0.66 (0.58–0.76)**	<0.001	0.97 (0.69–1.36)	0.854	1.32 (0.87–2.01)	0.190
3.4–4.0	**0.56 (0.43–0.72)**	<0.001	**0.46 (0.36–0.59)**	<0.001	**0.63 (0.53–0.73)**	<0.001	0.95 (0.65–1.40)	0.801	1.01 (0.63–1.61)	0.968
4.1–4.8	**0.60 (0.43–0.82)**	0.002	**0.53 (0.40–0.70)**	<0.001	**0.66 (0.55–0.80)**	<0.001	0.82 (0.53–1.27)	0.381	1.03 (0.62–1.73)	0.899
≥4.9	**0.48 (0.34–0.70)**	<0.001	**0.45 (0.32–0.63)**	<0.001	**0.68 (0.55–0.85)**	<0.001	1.12 (0.71–1.76)	0.638	1.03 (0.57–1.84)	0.930

*Adjusted for: oral medication for hyperlipidemia (yes/no/not applicable), age (numeric), sex (male/female), race (chinese/malay/indian/others), history of diabetes (yes/no), history of hypertension (yes/no), smoking status (never/former/current), history of AMI/CABG/PTCA (yes/no), BMI (numeric), Killip class on admission (1/2/3/4), CPR in ambulance/ED (yes/no), random blood glucose within 72 h from onset (numeric), creatinine on admission (numeric), haemoglobin on admission (numeric), elevated first troponin within 72 h from MI onset (yes/no), left ventricular ejection fraction <50% during hospitalization (yes/no).

### STEMI Patients

**Figure 1:**
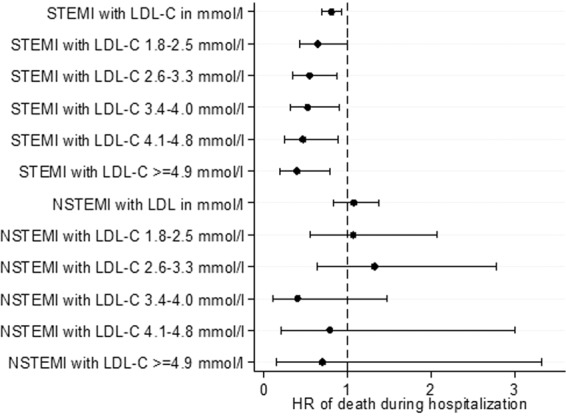
Forrest plots showing adjusted odds ratio of death during hospitalization across different LDL-C levels for patients with ST-elevation myocardial infarction (STEMI) and non ST-elevation myocardial infarction (NSTEMI).

**Figure 2:**
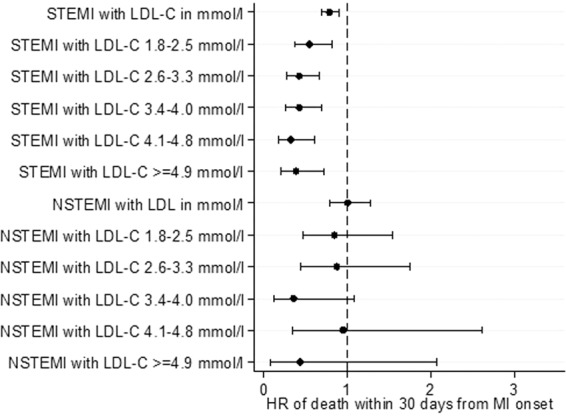
Forrest plots showing adjusted odds ratio of death within 30 days of myocardial infarction (MI) onset across different LDL-C levels for patients with ST-elevation myocardial infarction (STEMI) and non ST-elevation myocardial infarction (NSTEMI).

**Figure 3:**
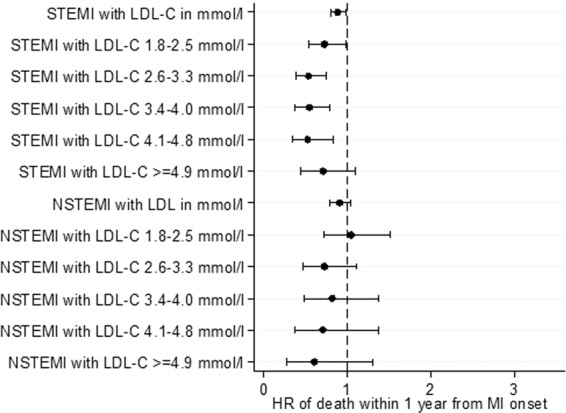
Forrest plots showing adjusted odds ratio of death within 1 year from MI onset across different LDL-C levels for patients with ST-elevation myocardial infarction (STEMI) and non ST-elevation myocardial infarction (NSTEMI).

**Figure 4:**
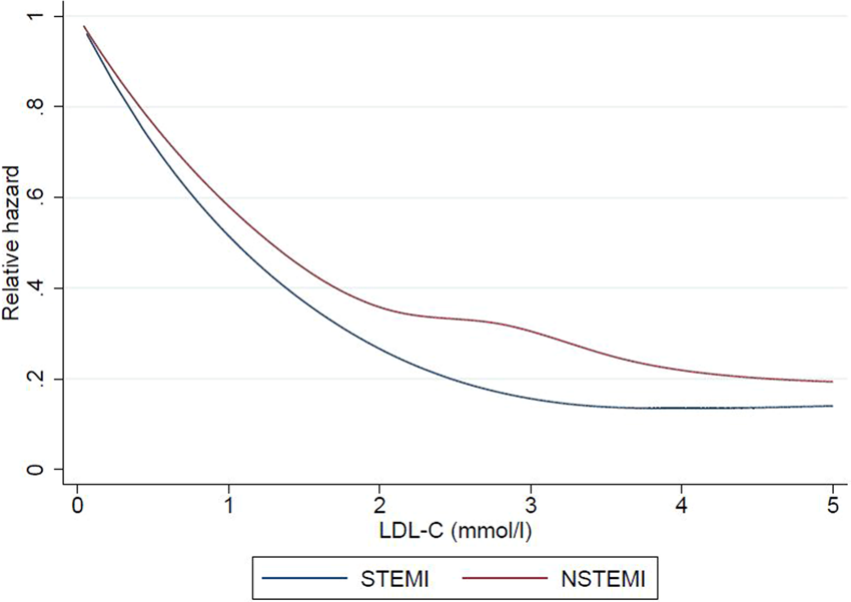
Restricted cubic spline showing the relationship between LDL-C levels for patients with ST-elevation myocardial infarction (STEMI) and non ST-elevation myocardial infarction (NSTEMI) and the relative hazard for death during hospitalization (**A**), at 30-days (**B**) and at 1-year (**C**) from MI.

**Figure 5:**
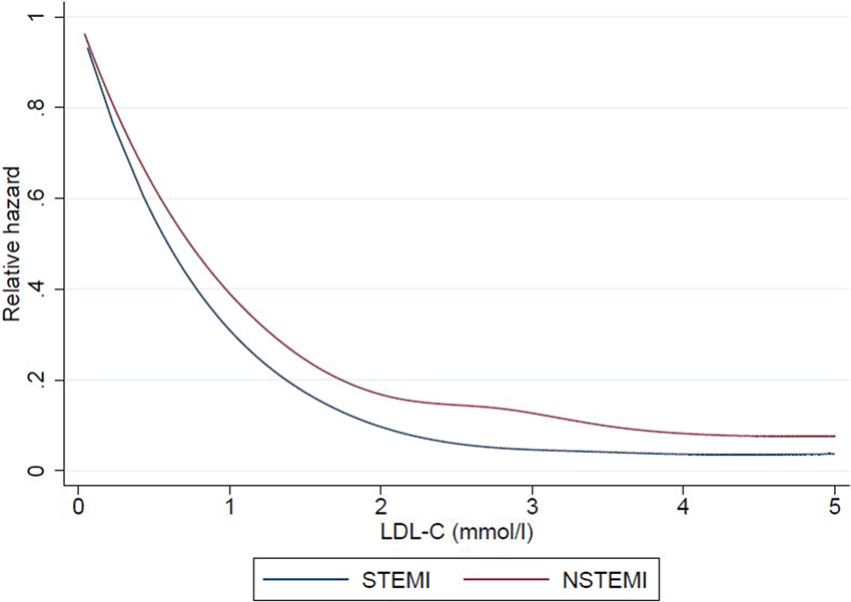
Restricted cubic spline showing the relationship between LDL-C levels for patients with ST-elevation myocardial infarction (STEMI) and non ST-elevation myocardial infarction (NSTEMI) and the relative hazard for death at 30-days from MI.

**Figure 6:**
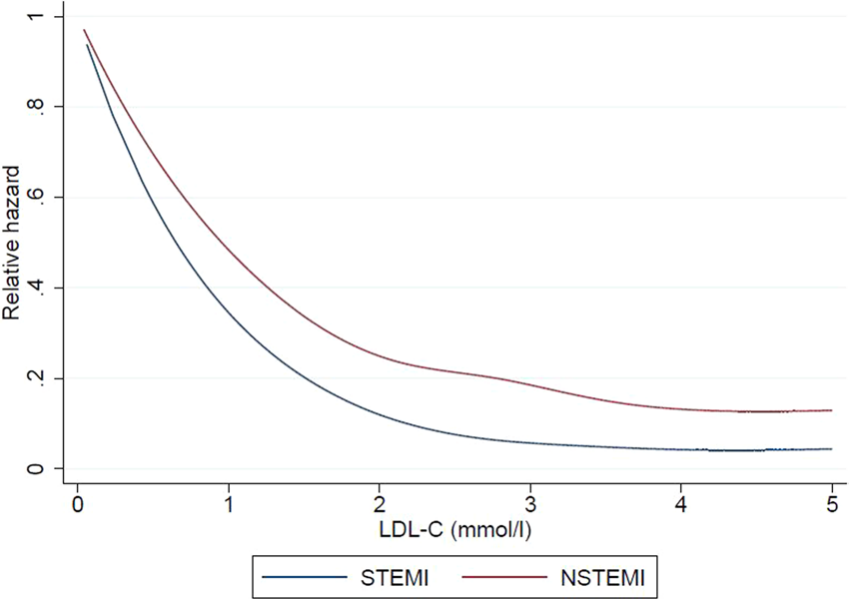
Restricted cubic spline showing the relationship between LDL-C levels for patients with ST-elevation myocardial infarction (STEMI) and non ST-elevation myocardial infarction (NSTEMI) and the relative hazard for death at 1-year from MI.

We next examined the correlations of lipid levels with the primary and secondary outcomes, for both STEMI and NSTEMI patients. Of note, in terms of primary outcomes for STEMI patients, a higher LDL-C was inversely correlated with a lower risk of death during hospitalization, at 30 days and at 1 year on both unadjusted and adjusted analyses (Figs. 1 to 6 and Supplementary Table 4). In terms of secondary outcomes for STEMI patients on unadjusted analysis, a higher LDL-C was similarly inversely correlated with a lower risk of rehospitalization for HF and AMI within 1 year from hospitalization discharge. After adjustment, the observed lipid paradox for the secondary outcomes of HF and AMI hospitalizations within 1 year from AMI discharge and higher LDL-C levels was no longer present. The significant variables after adjustment for HF hospitalizations were use of oral medications for hyperlipidemia, older age, Malay and Indian ethnicities, history of diabetes mellitus, history of hypertension, history of AMI/CABG/PCI, higher Killip class on admission, elevated troponin levels, longer symptom-to-balloon time and depressed LVEF. The significant variables after adjustment for AMI hospitalizations were driven by female sex, Malay, Indian and other ethnicities, smoking status and history of AMI/CABG/PCI. There was a similar relationship between a lower LDL-C and worse secondary outcomes for hospitalization for HF and AMI on unadjusted analysis but these correlations did not exist after adjustment (Supplementary Table 4).

There was an association between higher TC levels with worse primary outcomes on unadjusted and adjusted analysis for the primary outcomes but not for the secondary outcomes of interest (Supplementary Table 5).

For HDL-C, there was an association between lower HDL-C levels and better primary outcomes on unadjusted analysis but only the primary outcome of death during hospitalization persisted after adjustment. There was no such correlation for the secondary outcomes (Table 3). For TG, there was no strong association between TG levels and primary or secondary outcomes (Supplementary Table 6).

**Table 3:** Unadjusted and adjusted analysis examining the correlations between high density lipoprotein cholesterol levels and the primary and secondary outcomes in ST elevation myocardial infarction patients who underwent percutaneous coronary intervention.

	All STEMI patients						All STEMI patients discharged alive			
	Death during hospitalization		Death within 30 days from MI onset		Death within 1 year from MI onset		Rehospitalization for HF within 1 year from MI discharge		Rehospitalization for MI within 1 year from MI discharge	
	HR (95% CI)	p	HR (95% CI)	p	HR (95% CI)	p	HR (95% CI)	p	HR (95% CI)	p
**Unadjusted**										
HDL-C in mmol/l	1.17 (0.89–1.53)	0.264	1.13 (0.87–1.47)	0.356	**1.24 (1.03–1.50)**	0.020	1.09 (0.80–1.48)	0.576	1.09 (0.75–1.58)	0.638
**Ordinal categories**										
>=1.6	1.00 (ref)		1.00 (ref)		1.00 (ref)		1.00 (ref)		1.00 (ref)	
1.0–1.5	**0.46 (0.33–0.64)**	<0.001	**0.41 (0.30–0.58)**	<0.001	**0.40 (0.31–0.52)**	<0.001	0.69 (0.44–1.09)	0.110	0.83 (0.46–1.51)	0.543
<1.0	**0.55 (0.39–0.76)**	<0.001	**0.52 (0.37–0.72)**	<0.001	**0.46 (0.36–0.60)**	<0.001	0.67 (0.43–1.06)	0.085	0.79 (0.43–1.43)	0.430
Adjusted*										
HDL-C in mmol/l	1.22 (0.86–1.73)	0.274	0.94 (0.62–1.42)	0.760	1.03 (0.76–1.38)	0.865	0.87 (0.56–1.37)	0.552	1.12 (0.73–1.70)	0.614
**Ordinal categories**										
>=1.6	1.00 (ref)		1.00 (ref)		1.00 (ref)		1.00 (ref)		1.00 (ref)	
1.0–1.5	**0.55 (0.34–0.87)**	0.011	**0.53 (0.34–0.84)**	0.007	**0.56 (0.39–0.79)**	0.001	0.79 (0.42–1.47)	0.449	1.34 (0.54–3.30)	0.530
<1.0	0.64 (0.40–1.04)	0.072	0.77 (0.48–1.24)	0.285	0.74 (0.52–1.06)	0.103	0.94 (0.50–1.78)	0.856	1.18 (0.47–2.96)	0.727

*Adjusted for: oral medication for hyperlipidemia (yes/no/not applicable), age (numeric), sex (male/female), race (chinese/malay/indian/others), history of diabetes (yes/no), history of hypertension (yes/no), smoking status (never/former/current), history of AMI/CABG/PTCA (yes/no), BMI (numeric), Killip class on admission (1/2/3/4), CPR in ambulance/ED (yes/no), random blood glucose within 72 h from onset (numeric), creatinine on admission (numeric), haemoglobin on admission (numeric), elevated first troponin within 72 h from MI onset (yes/no), left ventricular ejection fraction <50% during hospitalization (yes/no), anterior MI (yes/no), symptom-to-balloon time (numeric).

### NSTEMI Patients

There was an inverse correlation between LDL-C levels and the primary outcomes of risk of death during hospitalization, at 30 days and at 1 year as well as the secondary outcomes of rehospitalization for heart failure and myocardial infarction within 1 year on unadjusted analysis for NSTEMI patients but was not present after adjustment (Supplementary Table 7).

There was an inverse relationship between the primary outcomes and TC levels and the secondary outcomes of rehospitalization for HF and AMI, but these correlations did not exist after adjustment (Supplementary Table 8). Lower HDL-C levels appeared to increase the risk of death during hospitalization after adjustment in contrast to the trend demonstrated in STEMI patients (Table 4). There was no correlation between TG levels and primary and secondary outcomes (Supplementary Table 9).

**Table 4:** Unadjusted and adjusted analysis examining the correlations between high density lipoprotein cholesterol levels and the primary and secondary outcomes in non-ST elevation myocardial infarction patients who underwent percutaneous coronary intervention.

	All NSTEMI patients						All NSTEMI patients discharged alive			
	Death during hospitalization		Death within 30 days from MI onset		Death within 1 year from MI onset		Rehospitalization for HF within 1 year from MI discharge		Rehospitalization for MI within 1 year from MI discharge	
	HR (95% CI)	p	HR (95% CI)	p	HR (95% CI)	p	HR (95% CI)	p	HR (95% CI)	p
**Unadjusted**										
HDL-C in mmol/l	0.78 (0.42–1.44)	0.426	0.90 (0.50–1.62)	0.728	**1.26 (1.00–1.58)**	0.046	1.14 (0.93–1.39)	0.218	1.23 (0.83–1.81)	0.304
**Ordinal categories**										
>=1.6	1.00 (ref)		1.00 (ref)		1.00 (ref)		1.00 (ref)		1.00 (ref)	
1.0–1.5	1.75 (0.63–4.85)	0.284	1.16 (0.46–2.89)	0.752	0.78 (0.50–1.23)	0.290	0.98 (0.53–1.82)	0.952	1.10 (0.58–2.11)	0.766
<1.0	1.53 (0.55–4.27)	0.415	1.08 (0.43–2.71)	0.868	0.79 (0.50–1.25)	0.313	0.75 (0.40–1.40)	0.371	0.97 (0.51–1.86)	0.935
Adjusted*										
HDL-C in mmol/l	0.61 (0.26–1.39)	0.236	0.68 (0.33–1.37)	0.276	0.94 (0.69–1.28)	0.700	1.06 (0.81–1.38)	0.672	1.33 (1.12–1.58)	0.001
**Ordinal categories**										
>=1.6	1.00 (ref)		1.00 (ref)		1.00 (ref)		1.00 (ref)		1.00 (ref)	
1.0–1.5	6.57 (0.88–48.84)	0.066	3.51 (0.84–14.68)	0.086	1.54 (0.84–2.83)	0.159	1.41 (0.62–3.21)	0.411	1.08 (0.48–2.47)	0.846
<1.0	5.42 (0.73–40.54)	0.099	3.27 (0.77–13.84)	0.107	1.64 (0.89–3.03)	0.112	1.15 (0.50–2.63)	0.741	1.01 (0.44–2.35)	0.978

*Adjusted for: oral medication for hyperlipidemia (yes/no/not applicable), age (numeric), sex (male/female), race (chinese/malay/indian/others), history of diabetes (yes/no), history of hypertension (yes/no), smoking status (never/former/current), history of AMI/CABG/PTCA (yes/no), BMI (numeric), Killip class on admission (1/2/3/4), CPR in ambulance/ED (yes/no), random blood glucose within 72 h from onset (numeric), creatinine on admission (numeric), haemoglobin on admission (numeric), elevated first troponin within 72 h from MI onset (yes/no), left ventricular ejection fraction <50% during hospitalization (yes/no).

### Interaction between type of myocardial infarction and lipids

Further analyses for interaction between the type of MI and lipids demonstrated a significant interaction term after adjustment for HDL-C only for the outcome for death during hospitalization (p = 0.015) (Table 5). There was no significant interaction after adjustment for all other outcomes and lipid levels.

**Table 5:** P value for interaction between type of myocardial infarction and lipids.

	Death during hospitalization	Death within 30 days from MI onset	Death within 1 year from MI onset	Rehospitalization for HF within 1 year from MI discharge	Rehospitalization for MI within 1 year from MI discharge
**LDL-C**					
Unadjusted	0.400	0.317	**0.001**	0.056	0.495
Adjusted*	0.506	0.951	0.209	0.194	0.109
**TG**					
Unadjusted	0.910	0.612	0.948	0.158	**0.042**
Adjusted8	0.731	0.942	0.893	0.211	0.244
**TC**					
Unadjusted	0.081	**0.026**	**<0.001**	**0.018**	0.448
Adjusted*	0.401	0.589	0.111	0.148	0.215
**HDL-C**					
Unadjusted	**0.034**	0.057	**0.040**	0.333	0.694
Adjusted*	**0.015**	0.081	0.060	0.635	0.604

*Adjusted for: oral medication for hyperlipidemia (yes/no/not applicable), age (numeric), sex (male/female), race (chinese/malay/indian/others), history of diabetes (yes/no), history of hypertension (yes/no), smoking status (never/former/current), history of AMI/CABG/PTCA (yes/no), BMI (numeric), Killip class on admission (1/2/3/4), CPR in ambulance/ED (yes/no), random blood glucose within 72 h from onset (numeric), creatinine on admission (numeric), haemoglobin on admission (numeric), elevated first troponin within 72 h from MI onset (yes/no), left ventricular ejection fraction <50% during hospitalization (yes/no).

Abbreviations: HDL-C, high density lipoprotein cholesterol; HF, heart failure; LDL-C, low density lipoprotein cholesterol; MI, myocardial infarction; TC, total cholesterol; TG, triglycerides.

## Discussion

Our main study findings were as follows: 1. The lipid paradox for LDL-C exists for STEMI patients undergoing PCI for the primary outcomes of death during hospitalization, at 30 days and at 1 year, but not for NSTEMI patients i.e. a pseudo-paradox was present for NSTEMI patients; 2. The lipid paradox for TG levels for STEMI patients undergoing PCI did not exist in our study after adjustment i.e. a pseudo-paradox is present; 3. HDL-C levels trended towards a paradox for STEMI patients but not for NSTEMI patients, and there was significant interaction between the type of MI and HDL-C levels for the outcome of death during hospitalization.

A number of studies have investigated the lipid paradox in patients with acute coronary syndromes. These studies were done in acute coronary syndrome populations that involved STEMI and NSTEMI populations as a whole, but did not specifically compare between these 2 groups^11,13,26–28^. Cho *et al*. studied a population of AMI patients post-PCI in relation to 30-day and 1-year outcomes, but did not stratify between the STEMI and NSTEMI groups. In their study, they found that patients with higher LDL-C levels, except for patients with LDL-C > 160 mg/dL (>4.1 mmol/L), were related to better outcomes. However, they reported independent predictive factors of 12-month mortality being age, systolic blood pressure, acute myocardial infarction, LVEF, renal function, Killip class, N-terminal-pro-B-type natriuretic level and use of renin-angiotensin receptor blockers (RAB) use, and concluded that their observation was an apparent paradox due to confounding factors. In our study, we accounted for the above variables (except for biomarker and RAB use) and demonstrated that the lipid paradox persisted in the STEMI but not the NSTEMI population. Interestingly, while we did not account for RAB use, the duration required for RAB use to effect positive myocardial remodeling in post-AMI patients would require time^29^. We observed the lipid paradox being present even for LDL-C in STEMI patients during the index hospitalization for myocardial infarction, which would not have been a sufficient duration of time for RABs to exert their myocardial remodeling effects. As such, we believe that RAB use, while prognostically useful in the long-term, would not explain our short-term observation.

For triglyceride levels in ACS patients, Cheng *et al*. studied a cohort of STEMI patients who received primary PCI in a single tertiary referral hospital and found that the serum triglyceride level had an inverse relationship with in-hospital death and late outcomes^30^. They postulate that higher TG levels may have a role in infarct size stabilization, reducing the risk of arrhythmias. An alternative postulated explanation is that TG actually reflects nutritional status and a lower TG means that the body’s nutritional state is poorer and hence may halt the patient’s recovery from STEMI. We did not find the same results in our study cohort after adjustment, nor was there any major differences between STEMI or NSTEMI groups for TG levels. A possible explanation is that our study adjusted for more variables compared to the study by Cheng *et al*., and there might be an apparent paradox for TG in that study due to residual confounding.

STEMI patients have been described to have an increased pro-inflammatory state compared to NSTEMI patients^15,31^. Our study also supports the role of inflammation as the underlying factor in the lipid paradox, as we demonstrated a lipid paradox in STEMI patients but not NSTEMI patients. Furthermore, this may be contributed by the fact that the STEMI and NSTEMI patients have different clinical characteristics. STEMI patients in our population were more likely to be smokers – the latter contributes to a pro-inflammatory state^32^. A counterpoint to this argument would be that the subjects in our STEMI population were more likely to be on oral medications for hyperlipidemia, and it is known that statins exert a pleiotropic anti-inflammatory effect^33^. Statins result in lower TC, LDL-C and TG levels^34^. A study demonstrated the effect of statins on outcome modification in patients with low LDL-C levels. Oduncu *et al*. demonstratedthat patients with statin-induced low LDL-C on admission had better outcomes in STEMI and predict lower mortality, but patients with spontaneously low LDL-C without statin treatment predict higher mortality^35^. Similarly, they postulate that statin exert an anticoagulant, anti-platelet and anti-inflammatory effect. Those with spontaneously low LDL-C in their study were associated with increased inflammation as reflected by higher inflammatory markers (leukocyte count, neutrophil/lymphocyte ratio and C-reactive protein levels)^35^.

Building on this, with regards to HDL-C, patients with a lower HDL-C trended towards better outcomes for STEMI patients (HDL-C lipid paradox). On the contrary, a lower HDL-C trended towards worse outcomes for NSTEMI patients, although this was only statistically significant for death during hospitalization at a level of HDL-C between 1.0–1.5 mmol/L. Previous studies in AMI populations have demonstrated that a lower HDL-C leads to greater mortality in both STEMI^36^ and NSTEMI patients^37^. This observation may be due to the possibility of the presence of dysfunctional HDL-C, which has been described to be present in patients with coronary artery disease, obesity, diabetes mellitus and smokers^38^. It is increasingly recognized that the function and subclass of HDL-C needs to be considered above the plasma concentrations, as plasma concentrations alone cannot account for the epidemiological observations and lack of treatment efficacy when raising HDL-C levels^39–41^. Dysfunctional HDL-C has a reduced pro-oxidative effect and increased pro-inflammatory effect. NSTEMI and STEMI patients have different levels of inflammation present, and this difference in inflammatory process can modify HDL-C functionality^41^, thus potentially leading to the observations in our study. Also, STEMI patients in our population were less likely to have a history of AMI/CABG/PCI (a surrogate for CAD), have a lower body mass index and less diabetes mellitus although there were a higher proportion of smokers compared to NSTEMI. This difference in baseline characteristics may also account for differing levels of dysfunctional HDL-C and hence a better outcome in STEMI patients. Further study of HDL-C function and subfractionsin addition to levels in this population would be helpful in the future in understanding this observation. Unfortunately, we did not have information on inflammatory markers in our population including C-reactive protein and total white cell count, nor did we have compliance data to statin use and could not specifically examine inflammation as a factor, but this can be the focus of future studies.

There have been other studies performed to examine the lipid paradox in cardiac patients in non-MI settings. Authors have described the potential pathophysiological mechanisms of a low LDL-C in situations of increased inflammation such as in heart failure. They explain that increased intestinal edema leads to an increase in translocation of bacterial lipoprotein saccharides (LPS) from the intestines into the blood, which induces inflammatory markers such as tumour necrosis factor-alpha. Lipoproteins form micelles around the bacterial LPS to inactivate the bacterial components, hence accounting for lower LDL-C levels^42,43^. While our study did not examine the biological mechanisms of the lipid paradox in post-MI PCI patients, gut bacteria have been linked to myocardial infarction and this could be a postulated mechanism of action^44^. Further potential explanations for the lipid paradox in heart failure patients include statin pre-medication as well as poorer nutritional status^43^, which wasafactor we adjusted for in our study.

The lipid paradox has also been described in non-cardiac conditions. Amezaga Urruela *et al*. described active rheumatoid arthritis patients having lower lipid levels, and postulated that this may be due to an inflammatory process^45^. A similar inflammatory cytokine release is observed in acute pancreatitis in which the lipid paradox has also been observed^46^. The inflammatory hypothesis is postulated to contribute significantly to the underlying pathophysiology of AMI^47^; this inflammatory hypothesis has recently been reinforced in the landmark Canakinumab Anti-inflammatory Thrombosis Outcome Study (CANTOS) trial which studied the use of the orphan drug canakinumab to reduce the risk of developing cardiovascular events using anti-inflammatory therapy with interleukin-1β inhibition^48^.

Finally, it might be prudent for clinicians not to be unduly influenced by the low measured LDL-C levels and hence withhold essential statin treatment for patients with acute myocardial infarction. This warning has been mentioned previously for nephrology patients in which the authors argue that despite the presence of the lipid paradox, statins exert an anti-inflammatory pleotropic effect which makes them effective medications for cardiovascular risk reduction^49^. This is supported by the American and European lipid management guidelines which advocate for the use of high intensity statins in AMI patients regardless of LDL-C levels^23,24^.

### Strengths and limitations

To the best of our knowledge, this is the largest study examining the lipid paradox in an unselected population of patients that are post-MI and have undergone PCI. Our study does have some limitations. As this is a cross-sectional analysis of registry data, we could demonstrate associations but not causation. The SMIR did not collect data on liver function tests as hepatic dysfunction is one of the postulated reasons accounting for the lipid paradox for LDL-C. Nevertheless, we did have information on Killip class and used this as a surrogate for predicting the possibility of liver failure. The SMIR did not collect data on biomarker levels such as brain-natriuretic peptide, specific subfractions and functionality testing of HDL-C, information on doses of in-hospital or pre-hospital statin use, nor did it collect PCI-procedural specific details (e.g. complexity of lesions), which may have contributed to outcomes.

## Conclusion

The lipid paradox appears to exist for LDL-C and TC levels and outcomes of death during hospitalization, death at 30 days and death at 1 year for STEMI patients. In the NSTEMI patients there appears to be a lipid pseudo-paradox. There is a significant interaction between HDL-C, the type of myocardial infarction and the outcome of death during hospitalization. These observations deserve further investigation.

## Supplementary information

Supplementary Information.

## Data Availability

The datasets are property of National Registry of Diseases and collected primarily for internal use. De-identified data can be accessed for public health research after approval from the Institutional Review Board and Ministry of Health.

## References

[1] Moran AE (2014). The Global Burden of Ischemic Heart Disease in 1990 and 2010. Circulation.

[2] Jeppesen J, Hein HO, Suadicani P, Gyntelberg F (1997). Relation of High TG–Low HDL Cholesterol and LDL Cholesterol to the Incidence of Ischemic Heart Disease. Arteriosclerosis, Thrombosis, and Vascular Biology.

[3] Wadhera RK, Steen DL, Khan I, Giugliano RP, Foody JM (2016). A review of low-density lipoprotein cholesterol, treatment strategies, and its impact on cardiovascular disease morbidity and mortality. Journal of Clinical Lipidology.

[4] Stampfer MJ (1996). A prospective study of triglyceride level, low-density lipoprotein particle diameter, and risk of myocardial infarction. Jama.

[5] Nordestgaard BG, Benn M, Schnohr P, Tybjærg-Hansen A (2007). Nonfasting Triglycerides and Risk of Myocardial Infarction, Ischemic Heart Disease, and Death in Men and Women. Jama.

[6] Grundy, S. M.*et al*.2018AHA/ACC/AACVPR/AAPA/ABC/ACPM/ADA/AGS/APhA/ASPC/NLA/PCNA Guideline on the Management of Blood Cholesterol. *Circulation*, Cir0000000000000625, 10.1161/cir.0000000000000625 (2018).

[7] Wilson PW (1990). High-density lipoprotein, low-density lipoprotein and coronary artery disease. The American journal of cardiology.

[8] Tall AR, Rader DJ (2018). Trials and Tribulations of CETP Inhibitors. Circulation Research.

[9] Nozue T (2016). Low-Density Lipoprotein Cholesterol Level and Statin Therapy in Patients With Acute Myocardial Infarction (Cholesterol Paradox). Circulation Journal.

[10] Cheng K-H (2015). Lipid paradox in acute myocardial infarction-the association with 30-day in-hospital mortality. Crit Care Med.

[11] Reddy VS (2015). Relationship between serum low-density lipoprotein cholesterol and in-hospital mortality following acute myocardial infarction (the lipid paradox). Am J Cardiol.

[12] Wang TY (2009). Hypercholesterolemia paradox in relation to mortality in acute coronary syndrome. Clinical cardiology.

[13] Cho KH (2010). Low-density lipoprotein cholesterol level in patients with acute myocardial infarction having percutaneous coronary intervention (the cholesterol paradox). Am J Cardiol.

[14] Kumar A, Cannon CP (2009). Acute coronary syndromes: diagnosis and management. Mayo Clin Proc.

[15] Di Stefano R (2009). Inflammatory markers and cardiac function in acute coronary syndrome: difference in ST-segment elevation myocardial infarction (STEMI) and in non-STEMI models. Biomed Pharmacother.

[16] Rott D, Leibowitz D (2007). STEMI and NSTEMI are two distinct pathophysiological entities. European heart journal.

[17] Ho AFW (2019). Time‐Stratified Case Crossover Study of the Association of Outdoor Ambient Air Pollution With the Risk of Acute Myocardial Infarction in the Context of Seasonal Exposure to the Southeast Asian Haze Problem. Journal of the American Heart Association.

[18] Ho AF (2016). Emergency Medical Services Utilization among Patients with ST-Segment Elevation Myocardial Infarction: Observations from the Singapore Myocardial Infarction Registry. Prehosp Emerg Care.

[19] Office, N. R. o. D.Singapore Myocardial Infarction Registry Annual Report 2007–2013 (2014).

[20] Zheng H (2019). Ethnic Differences and Trends in ST-Segment Elevation Myocardial Infarction Incidence and Mortality in a Multi-Ethnic Population. Ann Acad Med Singapore.

[21] Thygesen, K.*et al*.Fourth Universal Definition of Myocardial Infarction (2018). *Journal of the American College of Cardiology*, 25285, 10.1016/j.jacc.2018.08.1038 (2018). 10.1016/j.jacc.2018.08.1038 30153967

[22] Tai ES (2017). Ministry of Health Clinical Practice Guidelines: Lipids. Singapore medical journal.

[23] Hoes AW (2016). 2016ESC/EAS Guidelines for the Management of Dyslipidaemias. European heart journal.

[24] Grundy, S. M.*et al*.2018AHA/ACC/AACVPR/AAPA/ABC/ACPM/ADA/AGS/APhA/ASPC/NLA/PCNA Guideline on the Management of Blood Cholesterol. *Circulation*0, CIR.0000000000000625, 10.1161/CIR.0000000000000625.

[25] Government, S.*Registration of Births and Deaths Act (CHAPTER 267)*, https://sso.agc.gov.sg/Act/RBDA1937 (1987).

[26] Cheng KH (2015). Lipid paradox in acute myocardial infarction-the association with 30-day in-hospital mortality. Crit Care Med.

[27] Pokharel Y (2017). Association of low-density lipoprotein pattern with mortality after myocardial infarction: Insights from the TRIUMPH study. J Clin Lipidol.

[28] Wang TY (2009). Hypercholesterolemia Paradox in Relation to Mortality in Acute Coronary Syndrome. Clinical Cardiology.

[29] Anavekar NS, Solomon SD (2005). Angiotensin II receptor blockade and ventricular remodelling. J Renin Angiotensin Aldosterone Syst.

[30] Cheng YT (2014). Lower serum triglyceride level is a risk factor for in-hospital and late major adverse events in patients with ST-segment elevation myocardial infarction treated with primary percutaneous coronary intervention- a cohort study. BMC Cardiovasc Disord.

[31] Habib SS, Kurdi MI, Al Aseri Z, Suriya MO (2011). CRP levels are higher in patients with ST elevation than non-ST elevation acute coronary syndrome. Arq Bras Cardiol.

[32] Bakhru A, Erlinger TP (2005). Smoking cessation and cardiovascular disease risk factors: results from the Third National Health and Nutrition Examination Survey. PLoS medicine.

[33] Antonopoulos AS, Margaritis M, Lee R, Channon K, Antoniades C (2012). Statins as anti-inflammatory agents in atherogenesis: molecular mechanisms and lessons from the recent clinical trials. Curr Pharm Des.

[34] Meor Anuar Shuhaili MFR (2017). Effects of Different Types of Statins on Lipid Profile: A Perspective on Asians. Int J Endocrinol Metab.

[35] Oduncu V (2013). The prognostic value of very low admission LDL-cholesterol levels in ST-segment elevation myocardial infarction compared in statin-pretreated and statin-naive patients undergoing primary percutaneous coronary intervention. International journal of cardiology.

[36] Ji MS (2015). Impact of low level of high-density lipoprotein-cholesterol sampled in overnight fasting state on the clinical outcomes in patients with acute myocardial infarction (difference between ST-segment and non-ST-segment-elevation myocardial infarction). Journal of cardiology.

[37] Duffy D, Holmes DN, Roe MT, Peterson ED (2012). The impact of high-density lipoprotein cholesterol levels on long-term outcomes after non-ST-elevation myocardial infarction. American heart journal.

[38] Cybulska B, Klosiewicz-Latoszek L (2014). The HDL paradox: what does it mean and how to manage low serum HDL cholesterol level?. Kardiologia polska.

[39] Vergani C, Lucchi T (2012). PlasmaHDLcholesterol and risk of myocardial infarction. The Lancet.

[40] Martin SS (2014). HDL cholesterol subclasses, myocardial infarction, and mortality in secondary prevention: the lipoprotein investigators collaborative. European heart journal.

[41] Ramirez A, Hu PP (2015). Low High-Density Lipoprotein and Risk of Myocardial Infarction. Clin Med Insights Cardiol.

[42] von Haehling S, Schefold JC, Springer J, Anker SD (2008). The cholesterol paradox revisited: heart failure, systemic inflammation, and beyond. Heart Fail Clin.

[43] Velavan P, Huan Loh P, Clark A, Cleland JG (2007). The cholesterol paradox in heart failure. Congest Heart Fail.

[44] Rogler G, Rosano G (2013). The heart and the gut. European heart journal.

[45] Amezaga Urruela M, Suarez-Almazor ME (2012). Lipid paradox in rheumatoid arthritis: changes with rheumatoid arthritis therapies. Curr Rheumatol Rep.

[46] Hong W (2018). Relationship between low-density lipoprotein cholesterol and severe acute pancreatitis (“the lipid paradox”). Ther Clin Risk Manag.

[47] Blankenberg S, Yusuf S (2006). The Inflammatory Hypothesis. Circulation.

[48] Ridker PM (2017). Antiinflammatory Therapy with Canakinumab for Atherosclerotic Disease. New England Journal of Medicine.

[49] Wan RK, Mark PB, Jardine AG (2007). The cholesterol paradox is flawed; cholesterol must be lowered in dialysis patients. Semin Dial.

