# Supplementary material for: The Lipid Paradox is present in ST-elevation but not in non-ST-elevation myocardial infarction patients: Insights from the Singapore Myocardial Infarction Registry
Source: Sci Rep. 2020 Apr 22;10:6799. doi: 10.1038/s41598-020-63825-8 (PMC7176706; doi:10.1038/s41598-020-63825-8)
Supplement: Supplementary file 1 — Supplementary Information. [file 41598_2020_63825_MOESM1_ESM.pdf]

Supplementary Data

The Lipid Paradox is present in ST-elevation but not in non-ST-elevation myocardial infarction patients: Insights from the Singapore Myocardial Infarction Registry

Ching-Hui Sia[1,2], Huili Zheng[3], Andrew Fu-Wah Ho[4,5,6], Heerajnarain Bulluck[7], Jun Chong[5,6], David Foo[8], Ling-Li Foo[3], Patrick Zhan Yun Lim[9], Boon Wah Liew[10], Huay-Cheem Tan[1], Tiong-Cheng Yeo[1,2], Terrance Chua[11], Mark Yan-Yee Chan[1,2], Derek J Hausenloy[2,5,6,12,13,14]

[1] Department of Cardiology, National University Heart Centre Singapore, Singapore
[2] Yong Loo Lin School of Medicine, National University of Singapore, Singapore
[3] Health Promotion Board, National Registry of Diseases Office, Singapore, Singapore
[4] SingHealth Duke-NUS Emergency Medicine Academic Clinical Programme, Singapore
[5] Cardiovascular & Metabolic Disorders Program, Duke-NUS Medical School, Singapore
[6] National Heart Research Institute Singapore, National Heart Centre Singapore, Singapore
[7] Norfolk and Norwich University Hospital, Norwich, United Kingdom
[8] Tan Tock Seng Hospital, Singapore, Singapore
[9] Khoo Teck Puat Hospital, Singapore, Singapore
[10] Changi General Hospital, Singapore
[11] Department of Cardiology, National Heart Centre Singapore, Singapore
[12] The Hatter Cardiovascular Institute, University College London, London, United Kingdom
[13] Cardiovascular Research Center, College of Medical and Health Sciences, Asia University, Taiwan
[14] Tecnologico de Monterrey, Centro de Biotecnologia-FEMSA, Nuevo Leon, Mexico

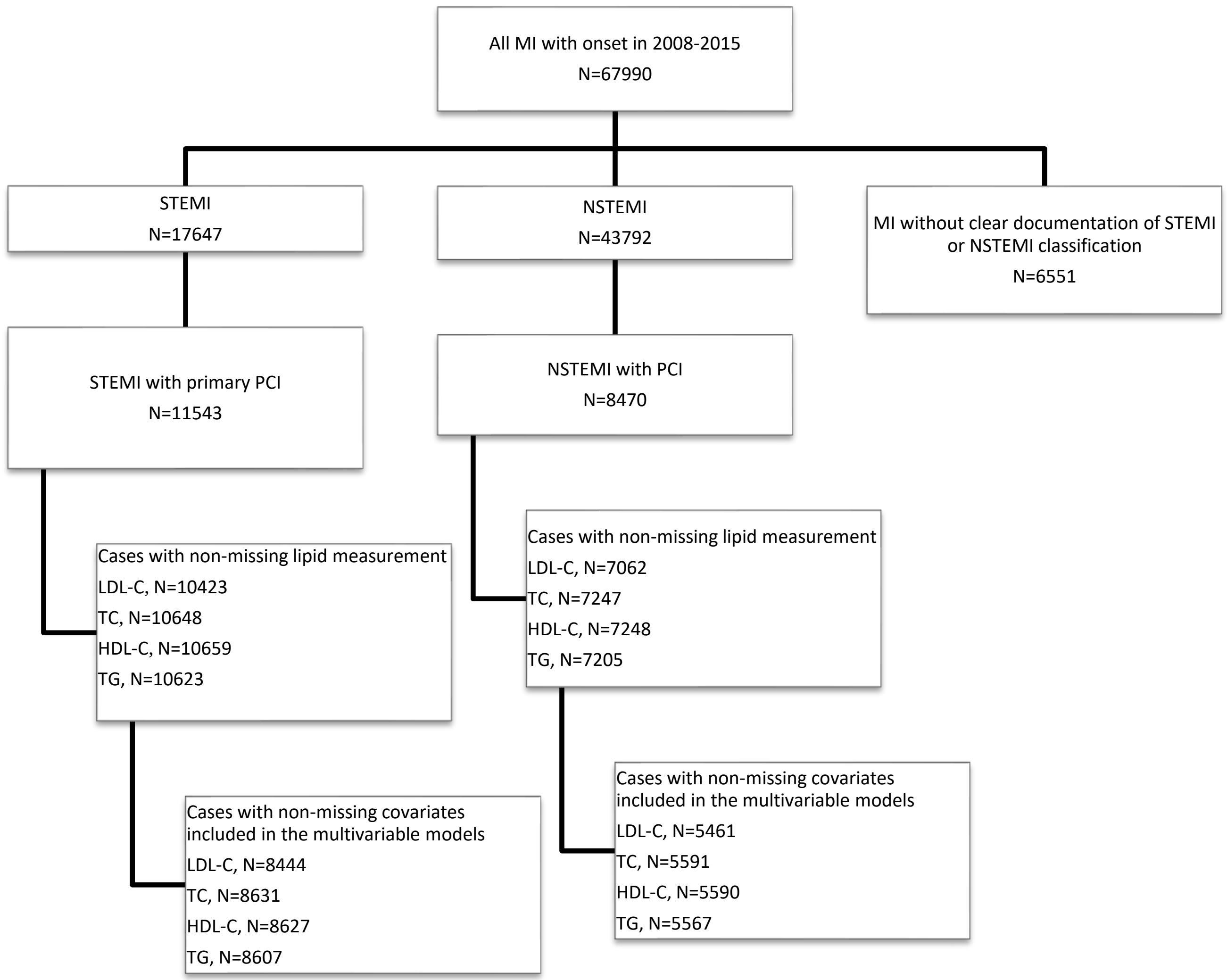

Supplementary Figure 1. Flow diagram of patients used for analysis.
Abbreviations: HDL-C, high-density lipoprotein cholesterol; LDL-C, low-density lipoprotein cholesterol; NSTEMI, non-ST segment elevation myocardial infarction; MI, myocardial infarction; PCI, percutaneous coronary intervention; STEMI, ST-segment elevation myocardial infarction; TC, total cholesterol; TG, triglycerides

	All STEMI+NSTEMI patients						All STEMI+NSTEMI patients discharged alive			
	Death during hospitalization		Death within 30 days from MI onset		Death within 1 year from MI onset		Rehospitalization for HF within 1 year from MI discharge		Rehospitalization for MI within 1 year from MI discharge	
	HR (95% CI)	p	HR (95% CI)	p	HR (95% CI)	p	HR (95% CI)	p	HR (95% CI)	p
**Unadjusted**										
TG in mmol/l	**0.80 (0.75-0.85)**	<0.001	**0.68 (0.64-0.73)**	<0.001	**0.68 (0.65-0.71)**	<0.001	**0.91 (0.84-0.99)**	0.030	1.00 (0.94-1.06)	0.913
Ordinal categories										
<1.7	1.00 (ref)		1.00 (ref)		1.00 (ref)		1.00 (ref)		1.00 (ref)	
1.7-2.2	**0.76 (0.66-0.88)**	<0.001	**0.61 (0.53-0.70)**	<0.001	**0.60 (0.55-0.66)**	<0.001	0.84 (0.67-1.05)	0.127	0.90 (0.70-1.16)	0.426
2.3-4.4	**0.59 (0.49-0.71)**	<0.001	**0.45 (0.38-0.53)**	<0.001	**0.44 (0.40-0.50)**	<0.001	0.84 (0.65-1.08)	0.173	1.06 (0.82-1.37)	0.680
>=4.5	**0.41 (0.24-0.70)**	0.001	**0.27 (0.17-0.45)**	<0.001	**0.35 (0.26-0.47)**	<0.001	0.52 (0.26-1.06)	0.072	1.02 (0.57-1.82)	0.944
**Adjusted***										
TG in mmol/l	0.98 (0.90-1.06)	0.595	0.96 (0.89-1.03)	0.242	0.96 (0.91-1.00)	0.076	0.98 (0.90-1.06)	0.559	0.93 (0.84-1.04)	0.189
Ordinal categories										
<1.7	1.00 (ref)		1.00 (ref)		1.00 (ref)		1.00 (ref)		1.00 (ref)	
1.7-2.2	1.02 (0.84-1.24)	0.856	0.96 (0.79-1.16)	0.675	0.94 (0.83-1.07)	0.344	0.87 (0.66-1.14)	0.311	**0.85 (0.62-1.15)**	0.296
2.3-4.4	0.85 (0.65-1.12)	0.245	0.89 (0.70-1.13)	0.336	0.87 (0.75-1.02)	0.096	1.07 (0.79-1.44)	0.680	0.79 (0.56-1.12)	0.188
>=4.5	0.70 (0.35-1.42)	0.328	0.61 (0.31-1.18)	0.142	0.86 (0.59-1.23)	0.402	0.54 (0.22-1.33)	0.180	0.80 (0.39-1.65)	0.551

Supplementary Table 1. Unadjusted and adjusted analysis examining the correlations between triglyceride levels and the primary and secondary outcomes in ST elevation myocardial infarction (STEMI) and Non-ST elevation myocardial infarction (NSTEMI) patients who underwent percutaneous coronary intervention

*adjusted for: oral medication for hyperlipidemia (yes/no/not applicable), age (numeric), sex (male/female), race (chinese/malay/indian/others), history of diabetes (yes/no), history of hypertension (yes/no), smoking status (never/former/current), history of AMI/CABG/PTCA (yes/no), BMI (numeric), Killip class on admission (1/2/3/4), CPR in ambulance/ED (yes/no), random blood glucose within 72h from onset (numeric), creatinine on admission (numeric), haemoglobin on admission (numeric), elevated first troponin within 72h from MI onset (yes/no), left ventricular ejection fraction <50% during hospitalization (yes/no)

	All STEMI+NSTEMI patients						All STEMI+NSTEMI patients discharged alive			
	Death during hospitalization		Death within 30 days from MI onset		Death within 1 year from MI onset		Rehospitalization for HF within 1 year from MI discharge		Rehospitalization for MI within 1 year from MI discharge	
	HR (95% CI)	p	HR (95% CI)	p	HR (95% CI)	p	HR (95% CI)	p	HR (95% CI)	p
**Unadjusted**										
TC in mmol/l	**0.78 (0.75-0.81)**	<0.001	**0.65 (0.63-0.68)**	<0.001	**0.69 (0.68-0.71)**	<0.001	0.94 (0.89-1.00)	0.054	0.96 (0.90-1.03)	0.234
Ordinal categories										
<5.2	1.00 (ref)		1.00 (ref)		1.00 (ref)		1.00 (ref)		1.00 (ref)	
5.2-6.1	**0.58 (0.50-0.68)**	<0.001	**0.43 (0.37-0.49)**	<0.001	**0.47 (0.43-0.52)**	<0.001	**0.77 (0.62-0.96)**	0.020	0.87 (0.69-1.09)	0.233
≥6.2	**0.52 (0.44-0.62)**	<0.001	**0.41 (0.35-0.48)**	<0.001	**0.48 (0.43-0.53)**	<0.001	0.94 (0.75-1.17)	0.570	0.95 (0.74-1.22)	0.693
**Adjusted***										
TC in mmol/l	**0.81 (0.77-0.86)**	<0.001	**0.78 (0.74-0.82)**	<0.001	**0.87 (0.84-0.90)**	<0.001	1.00 (0.93-1.07)	0.930	0.98 (0.90-1.07)	0.597
Ordinal categories										
<5.2	1.00 (ref)		1.00 (ref)		1.00 (ref)		1.00 (ref)		1.00 (ref)	
5.2-6.1	**0.73 (0.58-0.91)**	0.005	**0.67 (0.55-0.83)**	<0.001	**0.82 (0.72-0.93)**	0.003	0.93 (0.71-1.22)	0.610	0.95 (0.70-1.28)	0.744
≥6.2	**0.72 (0.56-0.93)**	0.011	**0.70 (0.55-0.88)**	0.002	**0.85 (0.74-0.99)**	0.035	1.12 (0.84-1.50)	0.441	0.92 (0.65-1.30)	0.634

Supplementary Table 2. Unadjusted and adjusted analysis examining the correlations between total cholesterol levels and the primary and secondary outcomes ST elevation myocardial infarction (STEMI) and Non-ST elevation myocardial infarction (NSTEMI) patients who underwent percutaneous coronary intervention
*adjusted for: oral medication for hyperlipidemia (yes/no/not applicable), age (numeric), sex (male/female), race (chinese/malay/indian/others), history of diabetes (yes/no), history of hypertension (yes/no), smoking status (never/former/current), history of AMI/CABG/PTCA (yes/no), BMI (numeric), Killip class on admission (1/2/3/4), CPR in ambulance/ED (yes/no), random blood glucose within 72h from onset (numeric), creatinine on admission (numeric), haemoglobin on admission (numeric), elevated first troponin within 72h from MI onset (yes/no), left ventricular ejection fraction <50% during hospitalization (yes/no)

	All STEMI+NSTEMI patients						All STEMI+NSTEMI patients discharged alive			
	Death during hospitalization		Death within 30 days from MI onset		Death within 1 year from MI onset		Rehospitalization for HF within 1 year from MI discharge		Rehospitalization for MI within 1 year from MI discharge	
	HR (95% CI)	p	HR (95% CI)	p	HR (95% CI)	p	HR (95% CI)	p	HR (95% CI)	p
**Unadjusted**										
HDL-C in mmol/l	0.89 (0.79-1.01)	0.075	0.90 (0.79-1.03)	0.120	**1.08 (1.00-1.17)**	0.037	0.88 (0.71-1.09)	0.239	0.93 (0.64-1.37)	0.720
Ordinal categories										
>=1.6	1.00 (ref)		1.00 (ref)		1.00 (ref)		1.00 (ref)		1.00 (ref)	
1.0-1.5	**0.82 (0.69-0.97)**	0.021	**0.70 (0.60-0.82)**	<0.001	**0.66 (0.59-0.73)**	<0.001	1.27 (0.88-1.83)	0.202	1.50 (0.97-2.33)	0.069
<1.0	0.92 (0.78-1.09)	0.319	**0.81 (0.69-0.95)**	0.011	**0.69 (0.62-0.77)**	<0.001	1.21 (0.84-1.75)	0.305	1.48 (0.96-2.29)	0.079
**Adjusted***										
HDL-C in mmol/l	**0.76 (0.63-0.93)**	0.006	**0.66 (0.54-0.80)**	<0.001	**0.76 (0.68-0.86)**	<0.001	0.90 (0.68-1.20)	0.469	1.20 (0.93-1.56)	0.162
Ordinal categories										
>=1.6	1.00 (ref)		1.00 (ref)		1.00 (ref)		1.00 (ref)		1.00 (ref)	
1.0-1.5	0.96 (0.76-1.22)	0.727	1.03 (0.82-1.29)	0.797	0.98 (0.85-1.13)	0.789	1.07 (0.68-1.68)	0.780	1.38 (0.79-2.42)	0.262
<1.0	1.19 (0.94-1.52)	0.156	**1.41 (1.12-1.78)**	0.004	**1.23 (1.06-1.43)**	0.008	1.16 (0.73-1.83)	0.534	1.24 (0.70-2.21)	0.462

Supplementary Table 3. Unadjusted and adjusted analysis examining the correlations between high density lipoprotein cholesterol levels and the primary and secondary outcomes in ST elevation myocardial infarction (STEMI) and Non-ST elevation myocardial infarction (NSTEMI) patients who underwent percutaneous coronary intervention

*adjusted for: oral medication for hyperlipidemia (yes/no/not applicable), age (numeric), sex (male/female), race (chinese/malay/indian/others), history of diabetes (yes/no), history of hypertension (yes/no), smoking status (never/former/current), history of AMI/CABG/PTCA (yes/no), BMI (numeric), Killip class on admission (1/2/3/4), CPR in ambulance/ED (yes/no), random blood glucose within 72h from onset (numeric), creatinine on admission (numeric), haemoglobin on admission (numeric), elevated first troponin within 72h from MI onset (yes/no), left ventricular ejection fraction <50% during hospitalization (yes/no)

	All STEMI patients						All STEMI patients discharged alive			
	Death during hospitalization		Death within 30 days from MI onset		Death within 1 year from MI onset		Rehospitalization for HF within 1 year from MI discharge		Rehospitalization for MI within 1 year from MI discharge	
	HR (95% CI)	p	HR (95% CI)	p	HR (95% CI)	p	HR (95% CI)	p	HR (95% CI)	p
**Unadjusted**										
LDL-C in mmol/l	**0.74 (0.68-0.81)**	<0.001	**0.63 (0.58-0.70)**	<0.001	**0.65 (0.60-0.70)**	<0.001	**0.82 (0.73-0.92)**	0.001	**0.86 (0.76-0.98)**	0.027
Ordinal categories										
<1.8	1.00 (ref)		1.00 (ref)		1.00 (ref)		1.00 (ref)		1.00 (ref)	
1.8-2.5	**0.58 (0.43-0.77)**	<0.001	**0.39 (0.29-0.51)**	<0.001	**0.45 (0.36-0.57)**	<0.001	**0.54 (0.37-0.77)**	0.001	0.81 (0.50-1.31)	0.383
2.6-3.3	**0.38 (0.28-0.52)**	<0.001	**0.24 (0.18-0.32)**	<0.001	**0.25 (0.20-0.32)**	<0.001	**0.45 (0.32-0.63)**	<0.001	**0.52 (0.32-0.84)**	0.007
3.4-4.0	**0.34 (0.24-0.48)**	<0.001	**0.19 (0.13-0.25)**	<0.001	**0.19 (0.15-0.24)**	<0.001	**0.30 (0.21-0.44)**	<0.001	**0.47 (0.29-0.77)**	0.003
4.1-4.8	**0.36 (0.24-0.54)**	<0.001	**0.18 (0.13-0.26)**	<0.001	**0.19 (0.14-0.25)**	<0.001	**0.35 (0.23-0.53)**	<0.001	**0.58 (0.35-0.98)**	0.040
≥4.9	**0.34 (0.22-0.55)**	<0.001	**0.22 (0.15-0.33)**	<0.001	**0.26 (0.19-0.35)**	<0.001	**0.41 (0.26-0.64)**	<0.001	**0.55 (0.30-0.99)**	0.048
**Adjusted***										
LDL in mmol/l	**0.81 (0.70-0.93)**	0.003	**0.79 (0.69-0.90)**	<0.001	**0.89 (0.81-0.99)**	0.024	1.02 (0.91-1.14)	0.700	0.85 (0.72-1.00)	0.052
Ordinal categories										
<1.8	1.00 (ref)		1.00 (ref)		1.00 (ref)		1.00 (ref)		1.00 (ref)	
1.8-2.5	0.65 (0.43-1.00)	0.051	**0.55 (0.37-0.82)**	0.004	**0.73 (0.54-0.99)**	0.044	0.67 (0.42-1.07)	0.096	0.91 (0.51-1.61)	0.742
2.6-3.3	**0.55 (0.35-0.87)**	0.010	**0.43 (0.28-0.67)**	<0.001	**0.54 (0.39-0.75)**	<0.001	0.83 (0.52-1.33)	0.442	0.70 (0.38-1.28)	0.246
3.4-4.0	**0.53 (0.32-0.90)**	0.019	**0.43 (0.26-0.70)**	0.001	**0.55 (0.38-0.80)**	0.002	0.80 (0.48-1.33)	0.382	0.54 (0.28-1.06)	0.073
4.1-4.8	**0.47 (0.25-0.89)**	0.021	**0.33 (0.18-0.61)**	<0.001	**0.53 (0.34-0.83)**	0.005	0.79 (0.46-1.39)	0.419	0.69 (0.34-1.40)	0.299
≥4.9	**0.40 (0.20-0.80)**	0.009	**0.39 (0.21-0.73)**	0.003	0.71 (0.45-1.10)	0.124	0.99 (0.54-1.82)	0.975	0.52 (0.22-1.21)	0.130

Supplementary Table 4. Unadjusted and adjusted analysis examining the correlations between low density lipoprotein cholesterol levels and the primary and secondary outcomes in ST elevation myocardial infarction patients who underwent percutaneous coronary intervention

*adjusted for: oral medication for hyperlipidemia (yes/no/not applicable), age (numeric), sex (male/female), race (chinese/malay/indian/others), history of diabetes (yes/no), history of hypertension (yes/no), smoking status (never/former/current), history of AMI/CABG/PTCA (yes/no), BMI (numeric), Killip class on admission (1/2/3/4), CPR in ambulance/ED (yes/no), random blood glucose within 72h from onset (numeric), creatinine on admission (numeric), haemoglobin on admission (numeric), elevated first troponin within 72h from MI onset (yes/no), left ventricular ejection fraction <50% during hospitalization (yes/no), anterior MI (yes/no), symptom-to-balloon time (numeric)

	All STEMI patients						All STEMI patients discharged alive			
	Death during hospitalization		Death within 30 days from MI onset		Death within 1 year from MI onset		Rehospitalization for HF within 1 year from MI discharge		Rehospitalization for MI within 1 year from MI discharge	
	HR (95% CI)	p	HR (95% CI)	p	HR (95% CI)	p	HR (95% CI)	p	HR (95% CI)	p
**Unadjusted**										
TC in mmol/l	**0.73 (0.67-0.79)**	<0.001	**0.61 (0.57-0.67)**	<0.001	**0.64 (0.60-0.69)**	<0.001	**0.81 (0.74-0.90)**	<0.001	**0.89 (0.80-1.00)**	0.050
Ordinal categories										
<5.2	1.00 (ref)		1.00 (ref)		1.00 (ref)		1.00 (ref)		1.00 (ref)	
5.2-6.1	**0.60 (0.46-0.79)**	<0.001	**0.49 (0.38-0.63)**	<0.001	**0.49 (0.40-0.60)**	<0.001	**0.65 (0.50-0.84)**	0.001	0.75 (0.55-1.02)	0.068
≥6.2	**0.59 (0.43-0.81)**	0.001	**0.46 (0.34-0.62)**	<0.001	**0.50 (0.40-0.63)**	<0.001	0.76 (0.57-1.00)	0.054	0.95 (0.68-1.31)	0.746
**Adjusted***										
TC in mmol/l	**0.81 (0.72-0.91)**	<0.001	**0.76 (0.68-0.86)**	<0.001	**0.88 (0.81-0.96)**	0.004	0.99 (0.90-1.10)	0.894	0.88 (0.76-1.02)	0.098
Ordinal categories										
<5.2	1.00 (ref)		1.00 (ref)		1.00 (ref)		1.00 (ref)		1.00 (ref)	
5.2-6.1	0.86 (0.58-1.27)	0.438	0.86 (0.59-1.24)	0.405	0.95 (0.72-1.25)	0.716	1.03 (0.73-1.44)	0.875	0.67 (0.43-1.06)	0.086
≥6.2	**0.59 (0.35-0.99)**	0.047	**0.53 (0.33-0.86)**	0.009	0.81 (0.58-1.13)	0.213	1.16 (0.80-1.67)	0.434	0.85 (0.54-1.36)	0.507

Supplementary Table 5. Unadjusted and adjusted analysis examining the correlations between total cholesterol levels and the primary and secondary outcomes in ST elevation myocardial infarction patients who underwent percutaneous coronary intervention

*adjusted for: oral medication for hyperlipidemia (yes/no/not applicable), age (numeric), sex (male/female), race (chinese/malay/indian/others), history of diabetes (yes/no), history of hypertension (yes/no), smoking status (never/former/current), history of AMI/CABG/PTCA (yes/no), BMI (numeric), Killip class on admission (1/2/3/4), CPR in ambulance/ED (yes/no), random blood glucose within 72h from onset (numeric), creatinine on admission (numeric), haemoglobin on admission (numeric), elevated first troponin within 72h from MI onset (yes/no), left ventricular ejection fraction <50% during hospitalization (yes/no), anterior MI (yes/no), symptom-to-balloon time (numeric)

	All STEMI patients						All STEMI patients discharged alive			
	Death during hospitalization		Death within 30 days from MI onset		Death within 1 year from MI onset		Rehospitalization for HF within 1 year from MI discharge		Rehospitalization for MI within 1 year from MI discharge	
	HR (95% CI)	p	HR (95% CI)	p	HR (95% CI)	p	HR (95% CI)	p	HR (95% CI)	p
**Unadjusted**										
TG in mmol/l	**0.80 (0.70-0.91)**	0.001	**0.67 (0.59-0.76)**	<0.001	**0.69 (0.62-0.76)**	<0.001	**0.86 (0.76-0.97)**	0.012	0.91 (0.81-1.03)	0.133
Ordinal categories										
<1.7	1.00 (ref)		1.00 (ref)		1.00 (ref)		1.00 (ref)		1.00 (ref)	
1.7-2.2	**0.75 (0.56-1.01)**	0.058	**0.56 (0.42-0.75)**	<0.001	**0.65 (0.52-0.81)**	<0.001	0.81 (0.61-1.08)	0.150	**0.67 (0.46-0.97)**	0.033
2.3-4.4	**0.63 (0.43-0.91)**	0.015	**0.41 (0.29-0.59)**	<0.001	**0.38 (0.29-0.52)**	<0.001	0.79 (0.57-1.08)	0.139	0.97 (0.68-1.38)	0.850
>=4.5	0.62 (0.28-1.39)	0.243	0.48 (0.23-1.01)	0.053	**0.47 (0.26-0.85)**	0.013	**0.37 (0.14-0.99)**	0.048	0.27 (0.07-1.10)	0.069
**Adjusted***										
TG in mmol/l	0.99 (0.85-1.15)	0.898	0.97 (0.84-1.13)	0.732	1.01 (0.91-1.12)	0.903	0.96 (0.85-1.07)	0.443	0.88 (0.73-1.05)	0.155
Ordinal categories										
<1.7	1.00 (ref)		1.00 (ref)		1.00 (ref)		1.00 (ref)		1.00 (ref)	
1.7-2.2	1.21 (0.78-1.86)	0.395	1.01 (0.66-1.54)	0.964	1.24 (0.93-1.66)	0.146	0.83 (0.58-1.20)	0.321	**0.57 (0.35-0.93)**	0.025
2.3-4.4	1.40 (0.80-2.46)	0.238	0.94 (0.55-1.60)	0.808	0.86 (0.58-1.27)	0.441	0.98 (0.65-1.46)	0.911	0.74 (0.46-1.19)	0.209
>=4.5	0.55 (0.08-3.95)	0.550	0.72 (0.18-2.96)	0.653	0.79 (0.29-2.15)	0.645	0.43 (0.11-1.68)	0.223	0.38 (0.10-1.53)	0.174

Supplementary Table 6. Unadjusted and adjusted analysis examining the correlations between triglyceride levels and the primary and secondary outcomes in ST elevation myocardial infarction patients who underwent percutaneous coronary intervention

*adjusted for: oral medication for hyperlipidemia (yes/no/not applicable), age (numeric), sex (male/female), race (chinese/malay/indian/others), history of diabetes (yes/no), history of hypertension (yes/no), smoking status (never/former/current), history of AMI/CABG/PTCA (yes/no), BMI (numeric), Killip class on admission (1/2/3/4), CPR in ambulance/ED (yes/no), random blood glucose within 72h from onset (numeric), creatinine on admission (numeric), haemoglobin on admission (numeric), elevated first troponin within 72h from MI onset (yes/no), left ventricular ejection fraction <50% during hospitalization (yes/no), anterior MI (yes/no), symptom-to-balloon time (numeric)

	All NSTEMI patients						All NSTEMI patients discharged alive			
	Death during hospitalization		Death within 30 days from MI onset		Death within 1 year from MI onset		Rehospitalization for HF within 1 year from MI discharge		Rehospitalization for MI within 1 year from MI discharge	
	HR (95% CI)	p	HR (95% CI)	p	HR (95% CI)	p	HR (95% CI)	p	HR (95% CI)	p
**Unadjusted**										
LDL-C in mmol/l	**0.72 (0.59-0.88)**	0.001	**0.56 (0.46-0.68)**	<0.001	**0.59 (0.53-0.66)**	<0.001	**0.77 (0.67-0.89)**	0.001	**0.73 (0.63-0.83)**	<0.001
Ordinal categories										
<1.8	1.00 (ref)		1.00 (ref)		1.00 (ref)		1.00 (ref)		1.00 (ref)	
1.8-2.5	0.67 (0.40-1.13)	0.135	**0.44 (0.27-0.73)**	0.001	**0.62 (0.46-0.83)**	0.001	0.71 (0.46-1.11)	0.131	**0.65 (0.43-1.00)**	0.048
2.6-3.3	**0.55 (0.32-0.96)**	0.035	**0.34 (0.20-0.57)**	<0.001	**0.30 (0.21-0.42)**	<0.001	**0.55 (0.35-0.86)**	0.008	**0.62 (0.41-0.95)**	0.026
3.4-4.0	**0.28 (0.12-0.65)**	0.003	**0.15 (0.07-0.31)**	<0.001	**0.25 (0.17-0.36)**	<0.001	**0.42 (0.25-0.68)**	0.001	**0.36 (0.22-0.59)**	<0.001
4.1-4.8	**0.25 (0.09-0.72)**	0.010	**0.15 (0.07-0.35)**	<0.001	**0.16 (0.10-0.27)**	<0.001	**0.27 (0.14-0.52)**	<0.001	**0.25 (0.13-0.46)**	<0.001
≥4.9	**0.20 (0.06-0.67)**	0.009	**0.07 (0.02-0.27)**	<0.001	**0.20 (0.12-0.35)**	<0.001	**0.54 (0.30-0.97)**	0.041	**0.35 (0.19-0.67)**	0.001
**Adjusted***										
LDL-C in mmol/l	1.08 (0.84-1.38)	0.550	1.01 (0.79-1.28)	0.957	0.91 (0.79-1.05)	0.183	1.10 (0.94-1.29)	0.238	1.16 (1.00-1.35)	0.052
Ordinal categories										
<1.8	1.00 (ref)		1.00 (ref)		1.00 (ref)		1.00 (ref)		1.00 (ref)	
1.8-2.5	1.07 (0.55-2.08)	0.850	0.85 (0.47-1.54)	0.593	1.05 (0.73-1.51)	0.782	1.04 (0.57-1.91)	0.897	1.25 (0.68-2.31)	0.475
2.6-3.3	1.33 (0.64-2.78)	0.443	0.88 (0.45-1.75)	0.724	0.73 (0.47-1.12)	0.148	1.27 (0.68-2.37)	0.446	**2.40 (1.26-4.58)**	0.008
3.4-4.0	0.41 (0.11-1.48)	0.175	0.36 (0.12-1.09)	0.070	0.82 (0.49-1.38)	0.453	1.68 (0.86-3.29)	0.129	1.78 (0.87-3.62)	0.113
4.1-4.8	0.79 (0.21-3.00)	0.730	0.95 (0.35-2.62)	0.923	0.71 (0.37-1.38)	0.314	0.96 (0.39-2.36)	0.929	1.45 (0.59-3.57)	0.422
≥4.9	0.70 (0.15-3.33)	0.650	0.44 (0.09-2.07)	0.300	0.61 (0.28-1.30)	0.199	1.80 (0.76-4.24)	0.181	2.22 (0.89-5.50)	0.086

Supplementary Table 7. Unadjusted and adjusted analysis examining the correlations between low density lipoprotein cholesterol levels and the primary and secondary outcomes in non-ST elevation myocardial infarction patients who underwent percutaneous coronary intervention

*adjusted for: oral medication for hyperlipidemia (yes/no/not applicable), age (numeric), sex (male/female), race (chinese/malay/indian/others), history of diabetes (yes/no), history of hypertension (yes/no), smoking status (never/former/current), history of AMI/CABG/PTCA (yes/no), BMI (numeric), Killip class on admission (1/2/3/4), CPR in ambulance/ED (yes/no), random blood glucose within 72h from onset (numeric), creatinine on admission (numeric), haemoglobin on admission (numeric), elevated first troponin within 72h from MI onset (yes/no), left ventricular ejection fraction <50% during hospitalization (yes/no)

	All NSTEMI patients						All NSTEMI patients discharged alive			
	Death during hospitalization		Death within 30 days from MI onset		Death within 1 year from MI onset		Rehospitalization for HF within 1 year from MI discharge		Rehospitalization for MI within 1 year from MI discharge	
	HR (95% CI)	p	HR (95% CI)	p	HR (95% CI)	p	HR (95% CI)	p	HR (95% CI)	p
**Unadjusted**										
TC in mmol/l	**0.69 (0.58-0.82)**	<0.001	**0.54 (0.45-0.64)**	<0.001	**0.59 (0.54-0.65)**	<0.001	**0.78 (0.69-0.88)**	<0.001	**0.76 (0.68-0.86)**	<0.001
Ordinal categories										
<5.2	1.00 (ref)		1.00 (ref)		1.00 (ref)		1.00 (ref)		1.00 (ref)	
5.2-6.1	**0.36 (0.18-0.75)**	0.007	**0.34 (0.19-0.61)**	<0.001	**0.36 (0.26-0.51)**	<0.001	**0.43 (0.29-0.65)**	<0.001	**0.57 (0.40-0.80)**	0.001
≥6.2	**0.27 (0.11-0.68)**	0.005	**0.19 (0.08-0.44)**	<0.001	**0.36 (0.25-0.52)**	<0.001	**0.66 (0.45-0.96)**	0.031	**0.54 (0.36-0.80)**	0.002
**Adjusted***										
TC in mmol/l	0.98 (0.78-1.23)	0.833	0.89 (0.72-1.11)	0.299	**0.89 (0.79-1.00)**	0.049	1.07 (0.93-1.22)	0.360	1.09 (0.96-1.24)	0.177
Ordinal categories										
<5.2	1.00 (ref)		1.00 (ref)		1.00 (ref)		1.00 (ref)		1.00 (ref)	
5.2-6.1	0.31 (0.09-1.05)	0.060	0.44 (0.17-1.13)	0.087	0.68 (0.43-1.06)	0.090	0.90 (0.56-1.47)	0.683	1.33 (0.87-2.04)	0.183
≥6.2	0.66 (0.22-1.96)	0.455	0.74 (0.30-1.83)	0.519	0.79 (0.49-1.28)	0.331	1.28 (0.75-2.19)	0.359	1.07 (0.61-1.90)	0.807

Supplementary Table 8. Unadjusted and adjusted analysis examining the correlations between total cholesterol levels and the primary and secondary outcomes in non-ST elevation myocardial infarction patients who underwent percutaneous coronary intervention

*adjusted for: oral medication for hyperlipidemia (yes/no/not applicable), age (numeric), sex (male/female), race (chinese/malay/indian/others), history of diabetes (yes/no), history of hypertension (yes/no), smoking status (never/former/current), history of AMI/CABG/PTCA (yes/no), BMI (numeric), Killip class on admission (1/2/3/4), CPR in ambulance/ED (yes/no), random blood glucose within 72h from onset (numeric), creatinine on admission (numeric), haemoglobin on admission (numeric), elevated first troponin within 72h from MI onset (yes/no), left ventricular ejection fraction <50% during hospitalization (yes/no)

	All NSTEMI patients						All NSTEMI patients discharged alive			
	Death during hospitalization		Death within 30 days from MI onset		Death within 1 year from MI onset		Rehospitalization for HF within 1 year from MI discharge		Rehospitalization for MI within 1 year from MI discharge	
	HR (95% CI)	p	HR (95% CI)	p	HR (95% CI)	p	HR (95% CI)	p	HR (95% CI)	p
**Unadjusted**										
TG in mmol/l	**0.64 (0.48-0.87)**	0.004	**0.47 (0.35-0.63)**	<0.001	**0.62 (0.53-0.71)**	<0.001	**0.75 (0.62-0.90)**	0.002	0.88 (0.77-1.01)	0.067
Ordinal categories										
<1.7	1.00 (ref)		1.00 (ref)		1.00 (ref)		1.00 (ref)		1.00 (ref)	
1.7-2.2	**0.49 (0.26-0.92)**	0.027	**0.45 (0.26-0.78)**	0.004	**0.45 (0.32-0.62)**	<0.001	**0.54 (0.36-0.79)**	0.002	0.78 (0.55-1.09)	0.149
2.3-4.4	**0.28 (0.11-0.70)**	0.006	**0.19 (0.08-0.43)**	<0.001	**0.41 (0.29-0.57)**	<0.001	**0.50 (0.33-0.75)**	0.001	**0.64 (0.44-0.94)**	0.022
>=4.5	0.38 (0.05-2.72)	0.333	0.18 (0.03-1.30)	0.090	**0.45 (0.21-0.95)**	0.037	0.44 (0.16-1.19)	0.106	1.17 (0.61-2.23)	0.635
**Adjusted***										
TG in mmol/l	0.90 (0.63-1.28)	0.549	0.74 (0.53-1.04)	0.083	0.92 (0.79-1.06)	0.252	0.91 (0.76-1.10)	0.334	0.93 (0.79-1.11)	0.437
Ordinal categories										
<1.7	1.00 (ref)		1.00 (ref)		1.00 (ref)		1.00 (ref)		1.00 (ref)	
1.7-2.2	1.14 (0.55-2.36)	0.718	1.18 (0.65-2.18)	0.584	0.80 (0.54-1.18)	0.266	0.73 (0.45-1.19)	0.208	1.03 (0.67-1.57)	0.899
2.3-4.4	0.85 (0.32-2.28)	0.753	0.51 (0.20-1.31)	0.162	0.85 (0.56-1.30)	0.459	0.79 (0.48-1.30)	0.352	0.65 (0.38-1.09)	0.105
>=4.5	Insufficient events		Insufficient events		1.25 (0.54-2.87)	0.602	0.63 (0.15-2.60)	0.523	1.47 (0.63-3.42)	0.374

Supplementary Table 9. Unadjusted and adjusted analysis examining the correlations between triglyceride levels and the primary and secondary outcomes in non-ST elevation myocardial infarction patients who underwent percutaneous coronary intervention

*adjusted for: oral medication for hyperlipidemia (yes/no/not applicable), age (numeric), sex (male/female), race (chinese/malay/indian/others), history of diabetes (yes/no), history of hypertension (yes/no), smoking status (never/former/current), history of AMI/CABG/PTCA (yes/no), BMI (numeric), Killip class on admission (1/2/3/4), CPR in ambulance/ED (yes/no), random blood glucose within 72h from onset (numeric), creatinine on admission (numeric), haemoglobin on admission (numeric), elevated first troponin within 72h from MI onset (yes/no), left ventricular ejection fraction <50% during hospitalization (yes/no)
